# Acute Myeloid Leukemia—Genetic Alterations and Their Clinical Prognosis 

**Published:** 2017-10-01

**Authors:** Francisco Alejandro Lagunas-Rangel, Venice Chávez-Valencia, Miguel Ángel Gómez-Guijosa, Carlos Cortes-Penagos

**Affiliations:** 1 Oncological Molecular Markers Laboratory, Graduate Studies Division, Faculty of Biological and Medical Sciences “Dr. Ignacio Chávez”, Universidad Michoacana de San Nicolás de Hidalgo, Morelia, Michoacán, México; 2 Nephrology Department, Regional General Hospital No. 1, Instituto Mexicano del Seguro Social, Morelia, Michoacán, México; 3 Hemostasy and Vascular Biology, Master in Health Sciences, Graduate Studies Division, Faculty of Biological and Medical Sciences “Dr. Ignacio Chávez”, Universidad Michoacana de San Nicolás de Hidalgo, Morelia, Michoacán, México; 4 Hematology Department, Regional General Hospital No. 1, Instituto Mexicano del Seguro Social, Morelia, Michoacán, México; 5 Oncological Molecular Markers Laboratory, Master in Health Sciences, Graduate Studies Division, Faculty of Biological and Medical Sciences “Dr. Ignacio Chávez”, Universidad Michoacana de San Nicolás de Hidalgo, Morelia, Michoacán, México

**Keywords:** AML, Mutations, Molecular marker, Cytogenetic, Risk groups

## Abstract

Acute myeloid leukemia (AML) is a group of hematological diseases, phenotypic and genetically heterogeneous, characterized by abnormal accumulation of blast cells in the bone marrows and peripheral blood. Its incidence rate is approximately 1.5 per 100,000 in infants younger than 1 year of age and 25 per 100,000 persons in octogenarians. Traditionally, cytogenetic markers are used to stratify patients in three risk categories: favorable, intermediate and unfavorable. However, the forecast stratification and the treatment decision for patients with normal karyotype shows difficulties due to the high clinical heterogeneity. The identification of several genetic mutations additional to classical molecular markers has been useful in identifying new entities. Nowadays, many different mutations and epigenetic aberrations have been implicated in the diagnostic, prognostic and treatment of AML. This review is focused on describing the most important molecular markers with implications for clinical practice.

## Introduction

 Acute myeloid leukemia (AML) is a group of hematological diseases, phenotypic and genetically heterogeneous, characterized by clonal expansion of myeloid precursors with diminished capacity for differentiation.^1^ AML represents 15 to 20% of acute leukemia cases in children and 80% in adults. AML is the predominant form of leukemia in neonatal and adult periods but represents a small fraction of cases during infancy and adolescence. There is a relatively small increase to approximately 1.5 cases per 100,000 in persons in their first year of life, representing congenital, neonatal, and infant AML. The incidence falls to a nadir of 0.4 new cases per 100,000 in persons over their first 10 years of life and then rises again to 1 case per 100,000 in persons in their second decade of life. Approximately from 25 years of age, the incidence increases exponentially to 25 cases per 100,000 in the octogenarian population.^2^

The first well-documented case of acute leukemia is attributed to Nikolaus Friedreich in 1857, but Wilhelm Ebstein was the first to use the term “acute leukämie” in 1889, referring to a disease with fast and fatal progression, thereby allowing clinical distinction between the acute and the chronic forms.^2^ The development of polychromatic stains by Paul Ehrlich in 1877 permitted the classification of leukemia into myeloid and lymphoid. In 1878, Ernst Neumann suggested for the first time that the bone marrow was the site of origin of leukemias and he used the term myelogene (myelogenous) leukemia. In 1900, Otto Naegeli distinguished between myeloblast and lymphoblast. Theodor Boveri in 1914 proposed the critical role played by chromosomal abnormalities in cancer development; however, it was not until 1960, with the Philadelphia Chromosome discovery, that the relationship between cytogenetic abnormalities and specific phenotypes of cancer became entrenched. Afterwards, with the conclusion of the Human Genome Project, scientists were able to identify the genes involved in recurrent cytogenetic abnormalities, as well as other genes associated with it. They were also able to understand more precisely the molecular pathology and come up with improved methods of diagnosis, prognosis and treatment for AML and other cancers.^2^^–^^4^

In the present work we briefly described the principal molecular markers implicated in the diagnosis, prognosis and treatment of AML with utility in the medical practice. 

Physiopathology

**Figure 1:**
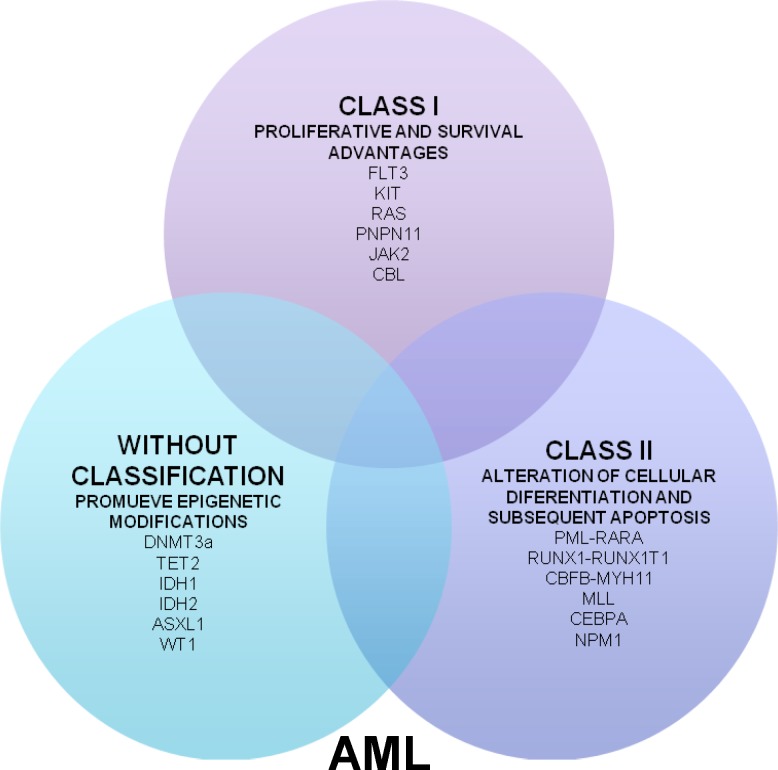
Model of cooperation between mutations associated with appearance of AML.

AML results from clonal transformation of hematopoietic precursors through the acquisition of chromosomal rearrangements and multiple gene mutations that confer a proliferative and survival advantage and impair hematopoietic differentiation.^5^^,^^6^ These key oncogenic events are often classified according to the two hits model proposed by Gilliland in 2001.^7^ This model hypothesizes that AML is the consequence of a collaboration between at least two broad classes of mutations, Class I mutations that confer proliferative and survival advantages, and Class II mutations that affect the processes of cell differentiation and apoptosis. However, recent studies using massively parallel sequencing technologies have identified other group of mutations that do not conform to any of the two classes; therefore, they have not been classified; however, these mainly promote epigenetic modifications (Figure 1) ^7^^–^^9^.

The model of the two hits hypothesizes that the AML is the result of collaboration between at least two types of mutations. The class I mutations confer proliferative and survival advantages, while class II mutations alter processes such as cellular differentiation and apoptosis. In addition, mutations that do not conform to any of the two classes have been found; therefore, they have not been classified; however, these mainly promote epigenetic modifications.

**Figure 2:**
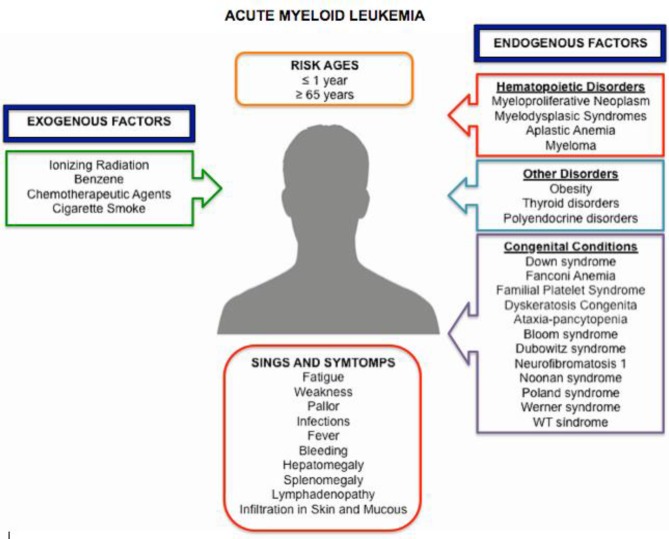
Etiology of AML. Many factors contribute to the development of AML, since exogenous and endogenous factors that associate and cause the appearance of the first signs and symptoms are generally diverse and nonspecific.

The basis of the leukemogenesis underlying in the non-lethal genetic damage in the case of AML includes a wide variety of factors that contribute in their development (Figura 2); however, the most important are high-dose radiation exposure, chronic, high-dose benzene exposure (≥40 parts per million [ppm]-years), chronic tobacco smoking, and chemotherapeutic agents (alkylating agents and topoisomerase II inhibitors principally). These exogen agents have the capacity of produce DNA damage through different mechanisms but principally by oxidative damage.^10^^–^^14^ Moreover, obesity is an endogenous agent that increases the risk of developing the disease; the precise mechanisms of it is still unclear but they may be related, in part, with the hyperinsulinemia, the insulin resistance, the elevated leptin levels, the decreased adiponectin levels and shortened telomeres found in these patients.^15^^,^^16^ On the other hand, AML can develop as a progression of other clonal disorder in hematopoietic stem cell (HSC) as a result of genomic instability and the acquisition of additional mutations.^2^ The main examples are myeloproliferative neoplasms (MPN) which increase the production of one or more types of blood cells and myelodysplastic syndromes (MDS) which stand out because of present defects in maturation that are associated with ineffective hematopoiesis.^17^ The first one, the NMP, is characterized by the presence of proteins tyrosine kinase mutated or with damages that activate their constitutive activity without the presence of the ligand, or well in signaling downstream effectors, which exemplifies class I mutations. Meanwhile, the SMD shows defects in key transcription factors for normal hematopoietic differentiation and apoptosis modulators, which resemble class II mutations.^7^ Thus, both disorders have a first hit, which makes them susceptible to develop AML if they are subjected to a second mutation. In addition, some hereditary conditions, such as those associated with DNA repair defects (i.e. Fanconi anemia), increase the risk of AML, susceptibility genes favoring a second mutation (i.e. familial platelet syndrome), tumor-suppressor defects (i.e. dyskeratosiscongenita), and unknown mechanisms, for example, ataxia-pancytopenia.^2^

Clinical presentation

The signs and symptoms of AML are diverse and nonspecific, but most of them are mainly attributed to the resulting cytopenia caused by leukemic infiltration of bone marrow. Normally, patients exhibit fatigue, bleeding, fever and infections due to decreased erythrocytes, platelets and functional leukocytes. Leukemic infiltration of other tissues include hepatomegaly, splenomegaly, lymphadenopathy, leukemia cutis and gingival also can affect the central nervous system and even an isolated mass of blasts (usually referred to as granulocytic sarcoma) can produce a variety of other symptoms.^2^^,^^17^^–^^19^

Diagnosis

For many years the diagnosis of AML was based solely on pathologic and cytological examination of bone marrow and peripheral blood smears; however, heterogeneity in the molecular mechanisms of this disease is manifested by morphological variability of the cells according to the type of lineage and degree of differentiation; this configured the basis for establishing certain subgroups. Initially proposed in 1976, the French-American-British group (FAB) established a classification method which divides the AML into eight different subtypes, according to the morphological appearance of blasts and their reactivity with histochemical stains, such as myeloperoxidase, black Sudan, and non-specific sterases α-naphthyl acetate and naphthyl butyrate. Additionally, some inmunological methods to analyze proteins on the cell surface and cytoplasmatic markers by flow cytometry have been incorporated into the classification criteria of the FAB. However, this classification does not always reflect the genetic and clinical diversity of the disease (Table 1A).^19^^–^^21^

**Table 1:** Classification systems of the FAB and WHO Acute Myeloid Leukemia

**A. Classification of Acute Myeloid Leukemia (FAB)**
M0. Acute myeloid leukemia without differentiation
M1. Acute myeloid leukemia with minimal differentiation
M2. Acute myeloid leukemia with differentiation
M3. Acute promyelocytic leukemia hipergranular or typical
M3v. Acute promyelocytic leukemia hipogranular
M4. Acute myelomonocytic leukemia
M4v. Acute myelomonocytic leukemia with bone marrow eosinophilia
M5. Acute monocytic leukemia
M6. Acute erythroid leukemia (Erythroleukemia)
M7. Acute Megacariocytic leukemia
**B. Classification of Acute Myeloid Leukema (OMS, 2008)**
AML with recurrent genetic abnormalities
Acute myeloid leukemia with t(8;21)(q22;q22); RUNX1-RUNX1T1
Acute myeloid leukemia with inv(16)(p13.1q22) or t(16;16)(p13.1;q22); CBFB-MYH11
Acute promyelocytic leukemia with t(15;17)(q22;q12); PML-RARA
Acute myeloid leukemia with t(9;11)(p22;q23); MLLT3-MLL
Acute myeloid leukemia with t(6;9)(p23;q34); DEK-NUP214
Acute myeloid leukemia with inv(3)(q21q26.2) or t(3;3)(q21;q26.2); RPN1-EVI1
Acute myeloid leukemia (megakaryoblastic) with t(1;22)(p13;q13); RBM15-MKL1
Acute myeloid leukemia with mutated NPM1
Acute myeloid leukemia with mutated CEBPA
Acute myeloid leukemia (AML) with myelodysplasia-related changes
Therapy-related myeloid neoplasms
Acute myeloid leukemia, NOS
Acute myeloid leukemia with minimal differentiation
Acute myeloid leukemia without maturation
Acute myeloid leukemia with maturation
Acute myelomonocytic leukemia
Acute monoblastic and monocytic leukemia
Acute erythroid leukemia
Acute megakaryoblastic leukemia
Acute basophilic leukemia
Acute panmyelosis with myelofibrosis

A way to recognize and classify different subgroups of AML through clinical, morphological and genetic correlation was proposed by the World Health Organization (WHO), which made a new classification that was updated in 2008. This classification has important differences with respect to the classification of the FAB. The blast threshold for the diagnosis of AML was modified from 30% to 20% in bone marrow or peripheral blood, and the categorization of cases LMA in a biological and clinical subgroup.

Three unique subgroups of AML are recognized by the WHO classification: AML with recurrent genetic abnormalities, AML with myelodysplastic-related changes and AML therapy-related myeloid neoplasms. Those cases that do not meet the criteria of these subgroups or in which genetic data cannot be obtained must be considered in the fourth subgroup: LMA without any other specification, which generally is based on the classification of the FAB and allows the universal application of the classification system (Table 1B) ^21^.

**Table 2:** Treatment system according to cytogenetic and molecular prognosis

**Patient**	**Risk Status**	**Induction**	**Post-Remission**
<60 years	Better-risk cytogenetics and/or molecular abnormalities	□ Clinical trial (preferred) □ Standard-dose cytarabine 100-200 mg/m^2^ continuous infusion x 7 days with idarubicin 12 mg/m^2^ or daunorubicin 90 mg/m^2^ x 3 days □ Standard-dose cytarabine 200 mg/m^2^ continuous infusion x 7 days with daunorubicin 60 mg/m^2^ x 3 days and cladribine 5 mg/m^2^ x 5 days □ High-dose cytarabine (HiDAC), 2 g/m^2^ every 12 hours x 6 days or 3 g/m^2^ every 12 h x 4 days with idarubicin 12 mg/m^2^ or daunorubicin 60 mg/m^2^ x 3 days (1 cycle)	□ Clinical trial □ HiDAC 3 g/m^2^ over 3 h every 12 h on days 1, 3, 5 x 3- 4 cycles □ 1 to 2 cycles of HiDAC-based consolidation followed by autologous HSCT
	Intermediate-risk cytogenetics and/or molecular abnormalities		□ Clinical trial □ Matched sibling or unrelated donor HSCT □ HiDAC 1.5-3 g/m^2^ over 3 h every 12 h on days 1, 3, 5 x 3-4 cycles □ 1 to 2 cycles of HiDAC-based consolidation followed by autologous HSCT
	Treatment-related disease or poor-risk cytogenetics and/or molecular abnormalities		□ Clinical trial □ Matched sibling or alternative donor HSCT
	APL	□ Idarubicin (5 mg/m^2^) on days 1-4 with cytarabine (500 mg/m^2^ per day) on days 1-4 and ATRA (45 mg/m^2^ per day) x 15 days □ Methotrexate (MTZ, 10 mg/m^2^ per day) on days 1-4 with ATRA (45 mg/m^2^ per day) x 15 days □ Idarubicin (12 mg/m^2^ per day) with cytarabine (500 mg/m^2^ per day) on days 1-2 and ATRA (45 mg/m^2^ per day) x 15 days	□ ATRA (45 mg/m^2^ per day) x 15 days with cada 3 meses with MTZ (15 mg/m^2^ x week each 3 months) and Mercaptopurine (50 mg/m^2^ per day)
≥60 años	Better-risk cytogenetics and/or molecular abnormalities	□ Clinical trial □ Standard-dose cytarabine (100-200 mg/m^2^ continuous infusion x 7 days) with idarubicin 12 mg/m^2^ or daunorubicin 45-90 mg/m^2^ x 3 days or mitoxantrone 12 mg/m^2^ □ Low-intensity therapy (subcutaneous cytarabine, 5-azacytidine, decitabine) □ Intermediate-intensity therapy (clofarabine)	□ Clinical trial □ Reduced-intensity HSCT □ Standard-dose cytarabine (100-200 mg/m^2^/day x 5- 7 d x 1-2 cycles) ± anthracycline (idarubicin or daunorubicin) □ Consider cytarabine1-1.5 g/m^2^/day x 4-6 doses x 1-2 cycles for patients with good performance status, normal renal function, better-risk or normal karyotype with favorable molecular markers □ Continue low-intensity regimens (5-azacytidine, decitabine) every 4-6 weeks until progression
	Intermediate-risk cytogenetics and/or molecular abnormalities	□ Clinical trial □ Low-intensity therapy (5-azacytidine, decitabine) □ Intermediate-intensity therapy (clofarabine) □ Standard-dose cytarabine (100-200 mg/m^2^ continuous infusion x 7 days) with idarubicin 12 mg/m^2^ or daunorubicin 45-60 mg/m^2^ x 3 days or mitoxantrone 12 mg/m^2^	□ Clinical trial □ Reduced-intensity HSCT in context of clinical trial □ Dosisestandar de Citabirina (100-200 mg/m^2^ en infusión continua por 7 días) con IDA (12 mg/m^2^por 3 diás) ó Daunorubicin (90 mg/m^2^por 3 días) o Mitoxantrone (12 mg/m^2^por 3 diás) □ 5-azacytidine, decitabine □ Clofarabine □ Best supportive care (hydroxyurea, transfusion support)
	Treatment-related disease or poor-risk cytogenetics and/or molecular abnormalities		
	APL	□ Idarubicin (5 mg/m^2^) on days 1-4 and ATRA (45 mg/m^2^ per day) x 15 days □ Methotrexate (10 mg/m^2^ per day) on days 1-3 with ATRA (45 mg/m^2^ per day) x 15 days □ Idarubicin (12 mg/m^2^) on day 1 and ATRA (45 mg/m^2^ per day) x 15 days	□ ATRA (45 mg/m^2^ per day) x 15 days with cada 3 meses with MTZ (15 mg/m^2^ x week each 3 months) and Mercvaptopurine (50 mg/m^2^ per day)

**Sources:** O’Donnell MR, Abboud CN, Altman J, et al. Acute myeloid leukemia. J NatlComprCancNetw. 2011;9(3):280-317. http://www.jnccn.org/content/9/3/280.full. Accessed October 22, 2014. Sanz-Alonso MA, Carreras-Pons E. Enfermedades del Sistema Mieloide. In: Rovira-Tarrats M, Sanz-Caballer J, eds. Manual Práctico de HematologíaClínica. 5th ed. Molins de Rei, España: Editorial Antares; 2015:149-164

Genetic and molecular landscape

Cytogenetics

The first proofs of the genetic basis of AML begins in cytogenetic analysis in which changes to the chromosomal level as translocations, deletions, insertions, inversions, monosomies, trisomies, polyploidy and other aberrations were detected. Usually one or more cytogenetic abnormalities are found in approximately 55% of patients with AML, and because of this configure a strong prognostic factor within the WHO classification.^22^

Currently, cytogenetic results allow the stratification of patients with AML into three classes, favorable, intermediate and unfavorable, according to clinical prognosis that is reported in the literature. So, patients with t(8;21) (q22;q22) [RUNX1/RUNX1T1], inv(16)(p13q22) [CBFB/MYH11] and t(15;17)(q24;q21) [PML/RARA] have a favorable prognosis with good response to treatment and complete remissions. On the other hand, patients with t(9;11)(p22;q23) [MLLT3/MLL] are considered to have intermediate prognosis, and patients with t(6;9)(p23;q34) [DEK/NUP214], inv(3)(q21q26) [RPN1/EVI1] and t(1;22)(p13;q13) [RBM15/MKL1] present an unfavorable prognosis due to the aggressiveness of the disease and poor response to treatment. These cytogenetic alterations produce fusion genes that encode aberrant proteins with altered functional characteristics.^23^^,^^24^

By the study of different mutations that accompany AML, it has been understood, though not entirely, the role of these critical genes. In the case of t(8;21) (q22;q22) and inv(16)(p13q22), whose fusion products are RUNX1/RUNX1T1 and CBFB/MYH11, respectively, are involved proteins of the family of core binding factors (CBF), which are required in the hematopoietic ontogeny and are key regulators in different steps of hematopoiesis. The CBF family consists of three subunits CBFA of DNA binding (RUNX1, RUNX2 and RUNX3) and a common subunit, CBFB, which do not interact with the DNA, but increase the affinity of the other subunits for this. Although the mechanisms by which these fusion genes contribute to the pathogenesis of leukemia are not fully understood, they have a dominant inhibitory activity on the target genes of wild CBF complex through recruitment of nuclear correpressor complex. Phenotypically, the t(8;21) (q22;q22) is associated predominantly with AML with maturation (FAB subtype M2), while the inv(16)(p13q22) is related to AML myelomonocytic with eosinophilia (FAB subtype M4v). Alterations on the KIT gene are associated as secondary mutations^23^^,^^25^.

On the other hand, the t(15;17)(q24;q21) is characteristic of AML promyelocytic (APL, FAB subtype M3). It involves the fusion of PML gene, a transcription factor and tumor suppressor that regulates cell cycle progression and induces cell death, and RARA, a nuclear retinoic acid receptor α which binds response elements to retinoic acid in promoters of many genes, resulting the fusion gene PML/RARA. Normally, RARA binds with the retinoid X receptor to form a heterodimer, which acts as a transcriptional repressor recruiting the nuclear corepressor complex histone deacetylase, thus facilitating the assembly of nucleosomes and silencing various promoters. The binding of its ligand (retinoic acid) causes a conformational change that results in transcriptional activation of genes required for the differentiation of promyelocytes. PML/RARA represses target promoters of the signaling chain in the same manner as RARA when it is not bound to its ligand; however, unlike the wild variant, it requires greater concentration of ligand to eliminate the repression because it maintains a more stable interaction with the corepressor complex and some methylases such as DNMT1 and DNMT3A. Furthermore, PML/RARA has important effects on apoptosis because it interferes in a negatively dominant manner with the function of wild PML and its regulation of p53. Thus, defects in apoptosis allow oncogene activation and persistence of genomic instability. Most patients with APL are sensitive to treatment with trans-retinoic acid (ATRA), which allows transcription of the DNA and, therefore, cell maturation. Secondary mutations involve mainly FLT3 gene^23^^,^^24^^,^^26^^,^^27^.

The t(9;11)(p22;q23) involves MLL and MLLT3 genes and is usually associated with leukemias with monocytic features as AML monocytic (FAB subtype M4) and AML myelomonocytic (FAB subtype M5). The fusion gene MLLT3-MLL is the most common rearrangement in AML of the MLL gene (although there are other less common rearrangements), which encodes a histone methyltransferase protein that by associating with protein complexes regulates transcription through the remodeling of chromatin. MLL is a positive regulator of the expression of the HOX genes and transcription factors involved in the development of multiple tissues, including the hematopoietic system. Rearrangements of MLL have several mechanisms to activate leukemogenic expression in the case of MLLT3-MLL where the domain of MLL that mediates H3K4 methylation (SET domain) is lost; the corresponding MLLT3 region contains domains for transcriptional control, interacts with DOT1L, another histone methyltransferase that methylates the lysine residue 79 in histone 3 (H3K79), and MENIN1, a transcriptional factor that binds to various promoters, thereby causing increased transcription of HOX genes, leading to cell proliferation and allowing reactivation, at least, in some aspects, of the cell self-renewal capacity^23^^,^^28^^,^^29^.

The balanced translocation t(6;9)(p23;q34), with the fusion gene DEK/NUP214 as marker, is related with leukemias with or without monocytic features and is often associated with basophilia and multilineage dysplasia; mainly it relates to AML with maturation (FAB subtype M2) and LMA myelomonocytic (FAB subtype M4) but may occur in some other phenotypes. DEK/NUP214 encodes a nucleoporin protein that acts as an aberrant transcription factor and additionally alters the nuclear transport to join with soluble transport factors. Mutations in FLT3 are related^23^.

The inv(3)(q21q26) involves the EVI1 gene, a transcription factor that has a specific expression pattern in HSC, which is essentially regulating self-renewal process. Notably, EVI1 regulates transcription factors such as GATA2, PBX1 and PLM; it can perform epigenetic modifications to silence certain genes by interacting with histone deacetylases and chromatin-modifying enzymes and activate other genes associated with acetyltransferases. RPN1 acts as such “enhancer” of the expression of EVI1, so the fusion gene causes increased proliferation and blocks cell differentiation inducing leukemic transformation. It presents any morphological pattern, with the exception of APL, but commonly presents as AML without maturation (FAB subtype M1), AML myelo-monocytic (M4) and AML megakaryoblastic (FAB subtype M7) ^23^^,^^30^.

The last recurrent cytogenetic abnormality in AML is the t(1;22)(p13;q13), which is associated with the fusion gene RBM15/MKL1, where the RNA binding motif from RBM15 is linking with the DNA binding motif involved with chromatin remodeling of MKL1. This fusion gene, therefore, modulates chromatin remodeling and differentiation associated with HOX and interferes in some ways with extracellular signaling. Mainly phenotype suggests an AML megakaryocytic (FAB subtype M7) ^23^.

Genetics

**Figure 3:**
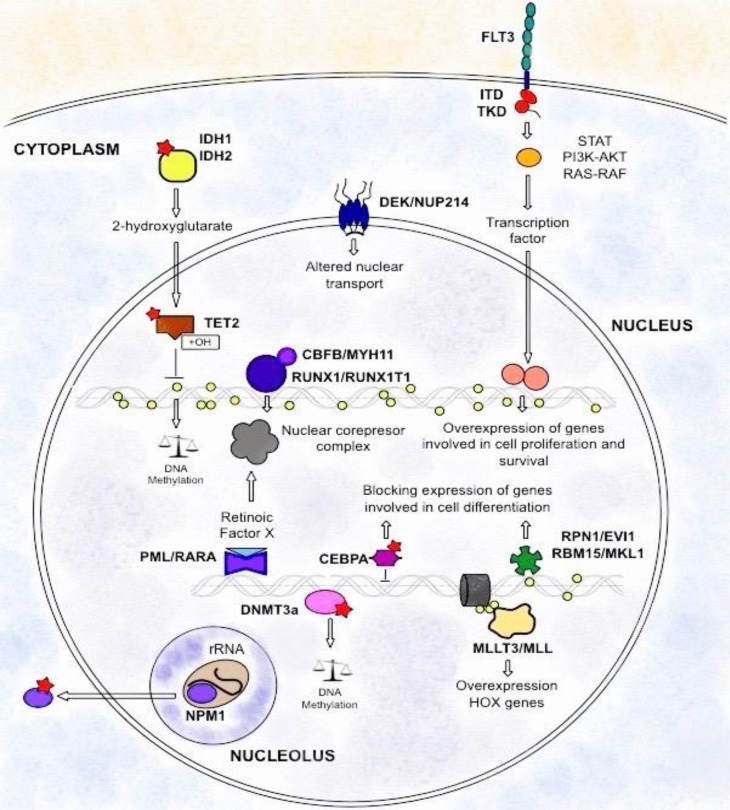
Scheme of the genetic-molecular landscape of AML. Cytogenetic factors involved t(8;21) (q22;q22) [RUNX1/RUNX1T1], inv(16)(p13q22) [CBFB/MYH11], t(15;17)(q24;q21) [PML/RARA], t(9;11)(p22;q23) [MLLT3/MLL], t(6;9)(p23;q34) [DEK/NUP214], inv(3)(q21q26) [RPN1/EVI1] and t(1;22)(p13;q13) [RBM15/MKL1]. Meanwhile, mutations in FLT3, NPM1 and CEBPA correspond to recurrent genetic abnormalities with prognostic value according to the WHO. The main epigenetic changes involve mutations in DNMT3a, TET2, IDH1 and IDH2 that modify DNA methylation patterns. Yellow circles indicate methyl groups, while the red stars represent mutations.

An important group of patients (approximately 45%) diagnosed with AML have a normal karyotype. These patients are classified with an intermediate clinical prognosis because clinically they do not have a reference marker and its biological origin is still unknown. Recently, with the development of methodologies of massive sequencing, new genetic mutations associated with acute myeloid leukemia have been identified. Some of the identified genes include KIT, FLT3, NPM1, CEBPA, RAS, WT1, BAALC, ERG, MN1, DNMT, TET2, IDH, ASXL1, PTPN11 and CBL. Of all these, WHO highlighting the related mutations in FLT3, NPM1 and CEBPα genes because they are associated with treatment response and progress of this disease (Figure 3) ^2^^,^^5^^,^^8^^,^^23^^,^^31^.

In 2008, the WHO published the updated classification of myeloid neoplasms; one of the major changes in this review is the incorporation of NPM1 and CEBPA mutations such as entities within the group of AML with recurrent genetic abnormalities. Mutations in FLT3 were not included as a separate entity because they are associated with several entities, but its significance should not be underestimated since its identification in patients with normal or with a chromosomal abnormality karyotype can determine the prognosis of leukemia^23^.

The FLT3 gene encodes a receptor tyrosine kinase (RTK) that plays a critical role in hematopoiesis and cell growth because it regulates diverse cellular processes such as proliferation, differentiation and apoptosis. It normally resides in the cell membrane as a monomer, with a configuration that prevents their activation^32^^,^^33^. The most common mutation in FLT3 involves an internal tandem duplication (ITD) between exons 14 and 15 in the juxtamembrane domain, which varies in length and position of patient to patient^33^. It has been suggested that the conformational change caused by the duplicate segment of FLT3-ITD is responsible for removing steric hindrance that normally blocks the dimerization without ligand stimulation, exposing various sites within the tyrosine kinase domains that induce its autophosphorylation^8^^,^^32^^,^^33^. The main impact of FLT3-ITD is its association with high blast accounts, increased risk of relapse and decreased survival. FLT3-ITD is especially frequent in patients with normal karyotype, t(15;17) (q22;q12) [PML-RARA] and t(6;9)(p23;q34) [DEK-NUP214]. Other mutations it is associated occur in NPM1 and DNMT3a.^32^^–^^35^ In contrast to wild FLT3 protein, FLT3-ITD active the STAT5 pathway significantly. STAT5 protein induces the gene expression of Cyclin D1, c-MYC and p21, which are important for cell proliferation. Moreover, the CEBPA and Pu.1 proteins involved in the regulation of differentiation in hematopoietic cells are significantly suppressed, suggesting their contribution to blocking differentiation^8^^,^^32^^–^^34^^,^^36^.

The second common type of mutation in FLT3 are missense mutations in exon 20 of the activation loop (A-loop) in the tyrosine kinase domain (TKD). Almost all these mutations involve the substitution of an aspartate with a tyrosine at codon 835 (D835Y) by a point mutation (GAT→TAT). Aspartate in the 835 position belongs to the domain DFG (Aspartate-Phenylalanine-Glycine) in the A-loop, playing a critical role in preventing efficient binding of ATP, being able to adopt an open (active) or closed (inactive) form. These mutations produce a conformational change in the protein, disrupting the energy balance required to stabilize the closed form, eliminating its autoinhibitory function that causes its constitutive activation. Other substitutions, deletions and insertions within this codon and other surroundings have also been identified^8^^,^^32^^,^^37^.

NPM1 is a protein that was originally identified as a phosphoprotein expressed at high levels in the granular region of the nucleolus. NPM resides principally in nucleoli, although it shuttles rapidly between the nucleus and cytoplasm, taking part in various cellular processes such as the transport of pre-ribosomal particles and ribosome biogenesis. The response to stress stimuli such as UV irradiation and hypoxia, the maintenance of genomic stability through the control of cellular ploidy and the participation in DNA-repair processes, and the regulation of DNA transcription through modulation of chromatin condensation and decondensation events, prevents protein aggregation in the nucleolus and participates in regulating the activity and stability of tumor suppressors such as p53 and ARF. NPM1 actually functions as histone chaperone that is capable of histone assembly, nucleosome assembly and increasing acetylation-dependent transcription^38^^,^^39^.

Mutations in the NPM1 gene are consistently heterozygous, appearing principally in exon 12, with a few exceptions reported in exon 11 and exon 9. Approximately 50 genetic variants have been described; however, 95% of cases occur in nucleotide position, 960 being the most common mutation, and the GTCT duplication of nucleotides at positions 956 to 959 is known as variant A. Regardless of the variant of the mutation, all generate modifications at the C terminus of the protein, generating an additional nuclear export domain rich in leucine and moreover the loss of the aromatic residues 288 and 290 that are crucial for nucleolar localization. For this reason, one of the distinguishing characteristics of mutations in NPM1 is its overexpression in the cytoplasm of leukemic cells with AML (NPM1c^+^)^40^^–^^42^.

NPM1 mutations are very stable; usually loss of mutation is associated with the change of karyotype, from normal to abnormal. Further NPM1 mutations are associated with a good response to therapy and five-year survival. The presence of NPM1 is significantly correlated with the presence of FLT3-ITD; in contrast, those mutations in tandem within the MLL gene are usually exclusive with NPM1. Phenotypically, they are associated with AML myelomonocytic (FAB subtype M4) and AML monocytic (FAB subtype M5) ^40^^,^^42^^,^^43^.

CEBPA, a transcription factor that plays a fundamental role in the early stages of myeloid differentiation, is particularly expressed in myelomonocytic cells, and is specifically overregulated during granulocytic differentiation. CEBPA results in two different transcripts, using two different AUG start sequences within the same reading frame; the first start sequence encodes an isoform of 42 KDa (p42), while the second start sequence encodes another isoform of 30 KDa (p30). Cells regulate the ratio p42/p30 through cell signaling triggered by rapamycin and protein kinase R as follows: under favorable growth conditions, the transcription initiation factors elF2α and eIF4E increase their activity, possibly by increasing the activity of c-MYC, and these act in promoting the transcription of p30 that initiates the process of cell proliferation. On the other hand, when there are low levels of eIF4E and elF2α, p42 transcription is promoted, inducing cellular differentiation^44^^,^^45^.

Mutations in CEBPα are point mutations that can affect transcription of the p42 variant, allowing overexpression of isoform p30, or affecting the leucine zipper region (bZIP) and the DNA binding domain, so that affects their interaction with DNA in the major groove and its dimerization and interaction with other proteins. Most patients have more than one mutation in CEBPα; the most common scenario is the combination of two mutations in different alleles, a mutation that blocks transcription of p42 and one in the bZIP, which are associated with favorable prognosis, as well as AML without maturation (FAB subtype M0) and AML with maturation (FAB subtype M2).^44^^,^^46^^,^^47^

Epigenetics

The epigenetic regulation allows modulation of transcription and gene expression without changing the genetic code. The two principal mechanisms of epigenetic regulation refers to post-transcriptional modifications of histones and DNA methylation and hydroxymethylation.^48^ The DNA methylation refers to the addition of a methyl group at the C5 position of the cytosine pyrimidine ring to form 5-methylcytosine (5mC), usually in the context of a CpG dinucleotide pair. Although 60 to 80% of individual CpGs are methylated, groups called CpG islands in regulatory regions are unmethylated. In general, a high DNA methylation is associated with silencing of gene expression. An aberrant methylation, mainly in promoters of tumor suppressor genes, has been observed in cancer. DNA methylation is mediated by the family methyltransferases which includes DNMT1, DNMT3A and DNMT3b; the first maintains preexisting methylation patterns, and the other two carry out de novo methylation in DNA. Moreover, proteins methylcytosine dioxygenase (TET1, TET2 and TET3) convert 5mC to 5-hydroxymethylcytosine (5hmC). The 5hmC is maintained by the DNMT1; however, this mark allows passive demethylation during cell division. Interestingly, somatic changes have been identified in DNMT3A, mainly resulting in the change of the arginine 882 of the catalytic domain with loss of methyl transferase activity; however, other mutations through gene have been observed. The mechanism by which mutations in DNMT3A trigger the development of leukemia are still unclear; however, it has been observed that mutations DNMT3A act dominantly negatively on the activity of wild DNMT3A. The prevalence of mutations in DNMT3a is associated with age, being common in patients of advanced age, with a frequency of 20 to 25% in de novo disease, and rare in pediatric patients. Furthermore, mutations in DNMT3a have been shown not to occur randomly, as they are never found in patients with t(15;17), inv(16) or t(8;21), as well as rearrangements of MLL; however, they are associated with mutations in FLT3-ITD and NPM1. It is important to note that the association between mutations in FLT3-ITD and DNMT3a may simply be a reflection of the concomitance of mutations in NPM1 and FLT3-ITD. Mutations in DNMT3a have demonstrated clinical relevance because they are allowed to stratify patients and generate new treatments. These mutations, generally, have shown an adverse time of survival in patients with intermediate cytogenetic prognosis. Phenotypically, mutations in DNMT3a are related with AML monocytic and AML myelo-monocytic (M5 and M4 subtypes FAB).^49^^,^^50^

The IDH proteins catalyze the oxidative decarboxylation of isocitrate to α-ketoglutarate in the tricarboxylic acid cycle. IDH1 is located in the cytoplasm and peroxisomes, while IDH2 is only in mitochondria. Mutations in IDH1 and IDH2 genes are found mainly in arginine residues highly conserved, in the case of IDH1, at residue 132, whereas in IDH2 at amino acids 140 and 172. Mutations in IDH1 and IDH2 contribute leukemogenesis because they acquire the ability to transform the α-ketoglutarate to 2-hydroxyglutarate, causing their accumulation, thus TET2 responsible for the formation of 5hmC, an enzyme that is Fe (II) and α-ketoglutarate dependent is inhibited. TET2 mutations occur in 7 to 23% of cases of AML and, although not clearly, have been associated with poor prognosis in patients with normal karyotype and favorable cytogenetic alterations^49^^,^^50^.

**Treatment**

Because AML is a heterogeneous group of disorders, they require different therapeutic interventions. In young patients, intensive chemotherapy is often used with cytarabine and anthracyclines, as well as other agents. Conventional therapy involves an induction dose of 100-200 mg/m^2^ of cytarabine continuous infusion for 7 days, accompanied with idarubicin at 12 mg/m^2^ for 3 days or daunorubicin at doses of 45-60 mg/m^2^ for 3 days, a therapy commonly referred to as 7 + 3. However, patients with cytogenetic or intermediate prognosis markers required more aggressive therapies with higher doses of cytarabine. On the other hand, elderly patients have a different biology because they are subject to conmorbilidades and have little tolerance for intensive chemotherapy. Generally, their treatment involves low-dose cytarabine accompanied by decitabine and 5-azacitidine or clofarabineaccompanied by other targeted therapies against FLT3, KIT, IDH1, and IDH2, among others^51^^,^^52^. APL has a different therapeutic regimen; schematically, three treatment options are currently proposed: conventional treatment with ATRA and chemotherapy, treatment with ATRA and chemotherapy reinforced with arsenic trioxide (ATO), and treatment with ATRA and ATO without or with minimal use of chemotherapy (Table 2) ^53^.

## CONCLUSION

 The heterogeneous clinical nature presented by patients with AML is simply a reflection of the molecular diversity of this disease, which results from a number of mutations affecting key points of processes of proliferation, survival, differentiation and apoptosis, or those that alter patterns of expression by epigenetic changes. It is important to note that although each patient presents a unique clinical condition, the interweaving of clinical, molecular and genetic features will establish a better view of the landscape that is located in front, allowing a glimpse of a more accurate and reliable forecast. The identification of molecular markers in AML has facilitated the discrimination of biologically and clinically distinct subgroups; furthermore, the correlation of mutational landscape with these and other “omic” data sets may further refine our understanding of AML biology, improve outcome prediction and treatment choices, such as the case of APL that the identification and study of the PML-RARA marker allowed the development of a specific treatment with ATRA. The understanding of the role played by gene mutations in leukemogenesis is likely to provide the basis for the development of new drugs and for a more rational use of the already existing anti-leukemic agents. Thus, several new drugs with targets in these molecular markers are being evaluated by different clinical trials, highlighting their importance. In this way, such as medical knowledge evolves, better treatment options that present greater specificity and fewer side effects will be created, gradually new discoveries will be added in the field, expanding the landscape of molecular alterations, new therapeutic targets, and changes in prognosis of AML.
